# Supplementary material for: Single cell and spatial transcriptomic analyses reveal microglia-plasma cell crosstalk in the brain during Trypanosoma brucei infection
Source: Nat Commun. 2022 Sep 30;13:5752. doi: 10.1038/s41467-022-33542-z (PMC9525673; doi:10.1038/s41467-022-33542-z)
Supplement: Supplementary file 1 — Supplementary Information [file 41467_2022_33542_MOESM1_ESM.pdf]

Supplementary Table 1.

List of primers used for RT-qPCR (all probes against *M. musculus* sequences)			
**Target**	**Sequence**	**Length (nt)**	**Melting temperature ($^{\circ}$C)**
**18S forward**	TGTGCCGCTAGAGGTGAAATT	21	57.9
**18S reverse**	TGGCAAATGCTTTCGCTTT	19	52.4
**IL-1β forward**	CCACAGACCTTCCAGGAGAATG	22	62.1
**IL-1 β reverse**	GTGCAGTTCAGTGATCGTACAGG	23	62.4
**TNFα forward**	ATGAGCACTGAAAGCATGATCC	22	58.4
**TNFα reverse**	GAGGGCTGATTAGAGAGAGGTC	22	62.1

Supplementary Table 2

List of antibodies used for immunofluorescence				
**Supplier**	**Cat. Number**	**Target**	**Clone**	**Dilution**
**Cell Signalling**	43279S	Arginase 1- Alexa fluor 647	D4E3M	1:100
**Miltenyi**	130-114-651	RE-Affinity CD68-PE	RAE886	1:50
**Santa Cruz**	sc-33673	GFAP-Alexa Fluor 488	2E1	1:100

Supplementary Table 3

**List of RNAscope probes used for smFISH**				
**Supplier**	**Cat. Number**	**Sequence**	**Channel**	**Dye used**
***Trypanosoma brucei* probes**				
**Biotechne**	1103198-C1	Tbr-*Gapdh*	Channel 1	Opal 520
**Biotechne**	1103208-C2	Tbr-*Pyk1*	Channel 2	Opal 650
**Biotechne**	1103218-C3	Tbr-*Pad2*	Channel 3	Opal 570
**Biotechne**	1103221-C4	Tbr-*Ep1*	Channel 4	Opal 540
***Mus musculus* probes**				
**Biotechne**	460181	Mm-*Cd79a*	Channel 1	Opal 520
**Biotechne**	314221-C2	Mm-*Cx3cr1*	Channel 2	Opal 650
**Biotechne**	317261-C3	Mm-*Il10*	Channel 3	Opal 570
**Biotechne**	517731-C4	Mm-*Il10ra*	Channel 4	Opal 540
**Biotechne**	414891-C3	Mm-*Tnfsf13b*	Channel 3	Opal 570
**Biotechne**	414871-C4	Mm-*Tnfrsf17*	Channel 4	Opal 540

Supplementary Table 4

**List of antibodies used for flow cytometry**				
**Supplier**	**Cat. Number**	**Target**	**Clone**	**Dilution**
Thermo	65-0865-14	Fixable viability dye eFluor 780	-	1:1,000
Biolegend	147712	CD45 PE	I3/2.3	2 μg/100 μl i.v.
Biolegend	103133	CD45 Brilliant Violet 421	30-F11	1:400
Biolegend	115555	CD19 Brilliant Violet 711	6D5	1:400
Biolegend	103225	B220 Alexa Fluor 488	RA3-6B2	1:400
Biolegend	142527	CD138 PE-Dazzle 594	281-2	1:400
Biolegend	101219	CD11b Alexa Fluor 488	M1/70	1:400
Biolegend	366507	BAFF APC	1D6	1:400
Biolegend	141727	CD206 Brilliant Violet 711	C068C2	1:400
Miltenyi	130-119-982	O4 APC	REA576	1:800
Miltenyi	130-123-284	ACSA-PE	IH3-18A3	1:800

Supplementary figure legends

Figure S1

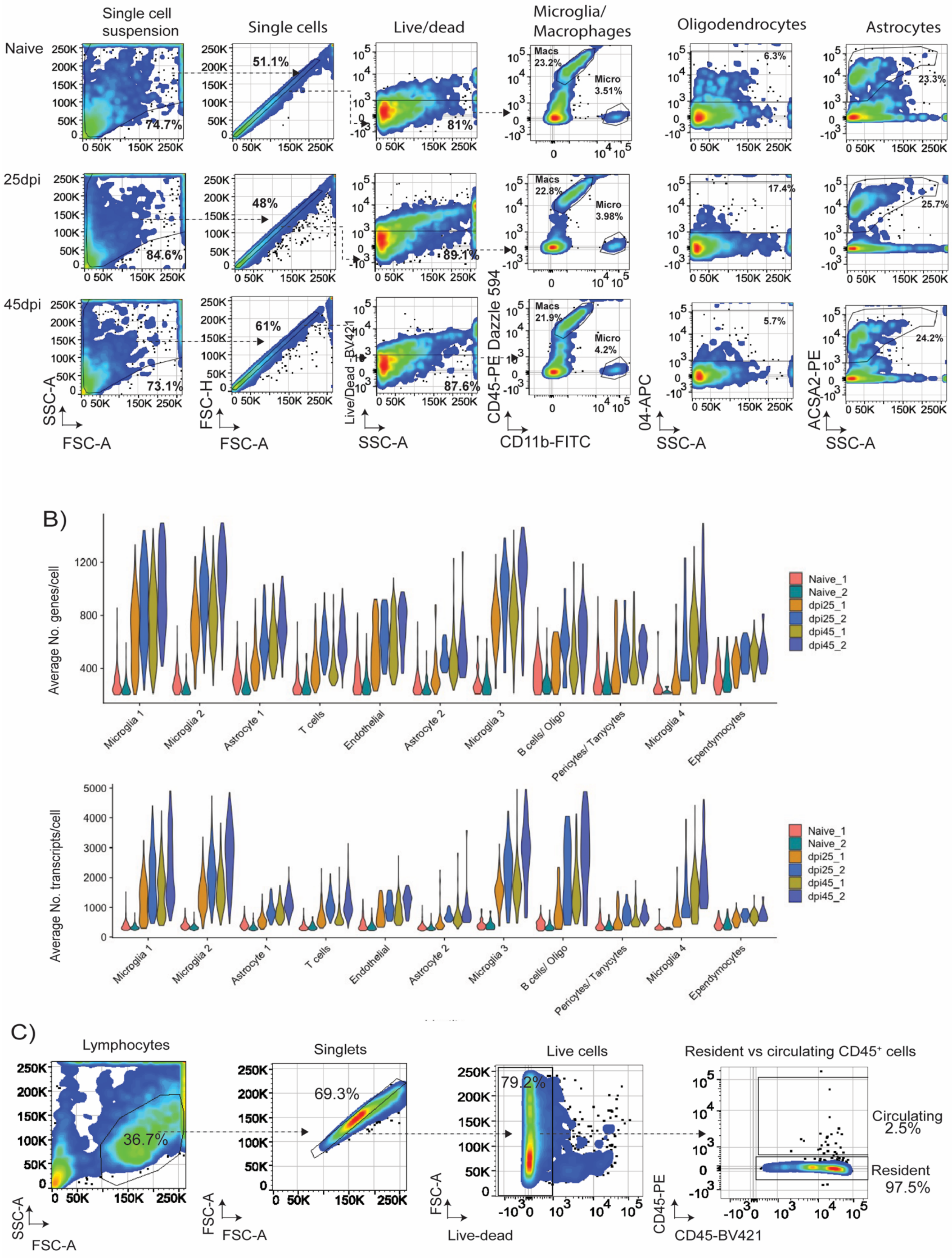
Single cell suspension
Single cells
Live/dead
Microglia/ Macrophages
Oligodendrocytes
Astrocytes
Naive
25dpi
45dpi
74.7%
51.1%
81%
Macs 23.2%
Micro 3.51%
6.3%
23.3%
84.6%
48%
89.1%
Macs 22.8%
Micro 3.98%
17.4%
25.7%
73.1%
61%
87.6%
Macs 21.9%
Micro 4.2%
5.7%
24.2%
SSC-A
FSC-A
FSC-H
Live/Dead -BV421
CD45-PE Dazzle 594
CD11b-FITC
04-APC
ACSA2-PE
B)
Average No. genes/cell
Average No. transcripts/cell
Naive_1
Naive_2
dpi25_1
dpi25_2
dpi45_1
dpi45_2
Microglia 1
Microglia 2
Astrocyte 1
T cells
Endothelial
Astrocyte 2
Microglia 3
B cells/ Oligo
Pericytes/ Tanycytes
Microglia 4
Ependymocytes
C)
Lymphocytes
Singlets
Live cells
Resident vs circulating CD45⁺ cells
36.7%
69.3%
79.2%
Circulating 2.5%
Resident 97.5%
Live-dead
CD45-PE
CD45-BV421

Supplementary Figure 1. Quality control of the hypothalamic scRNAseq datasets over the course of infection with *T. brucei*.
A) Representative flow cytometry analysis from naïve sample (top panel), and infected samples at 25 (middle panel) and 45 days post-infection (bottom panel), showing the relative proportion of macrophages (Cd45^{High} CD11b^{high}), microglia (CD11b^{High} CD45^{low}), oligodendrocytes (O4^{+}), and astrocytes (ACSA2^{+}) from the live cells gate. **B)** Average number of genes (top) and transcripts (bottom) per cell in the hypothalamic scRNAseq after filtering low quality cells, split by biological replicate. For normalisation, we accounted for differential gene and UMI counts using two independent approaches (SCT and STACAS). Both packages broadly identified the same cell populations. C) Top 25 most highly variable genes identified by *Scater*. The dotted line represents the median gene count. **D)** Gating strategy for the identification of brain resident CD45^{+} immune cells. Before processing, mice were inoculated i.v. with anti-CD45 antibody conjugated with PE, labelling all circulating CD45^{+} immune cells. After lymphocyte preparation from brain samples using Percoll gradient, samples were re-stained with anti-CD45 antibody conjugated with Brilliant Violet 421 labelling tissue resident CD45^{+} immune cells not previously exposed to anti-CD45-PE. The combination of these fluorophores allows to separate circulating from resident immune cells. This gating strategy was used to identify brain-resident plasma cells, as well as BAFF^{+} microglia.

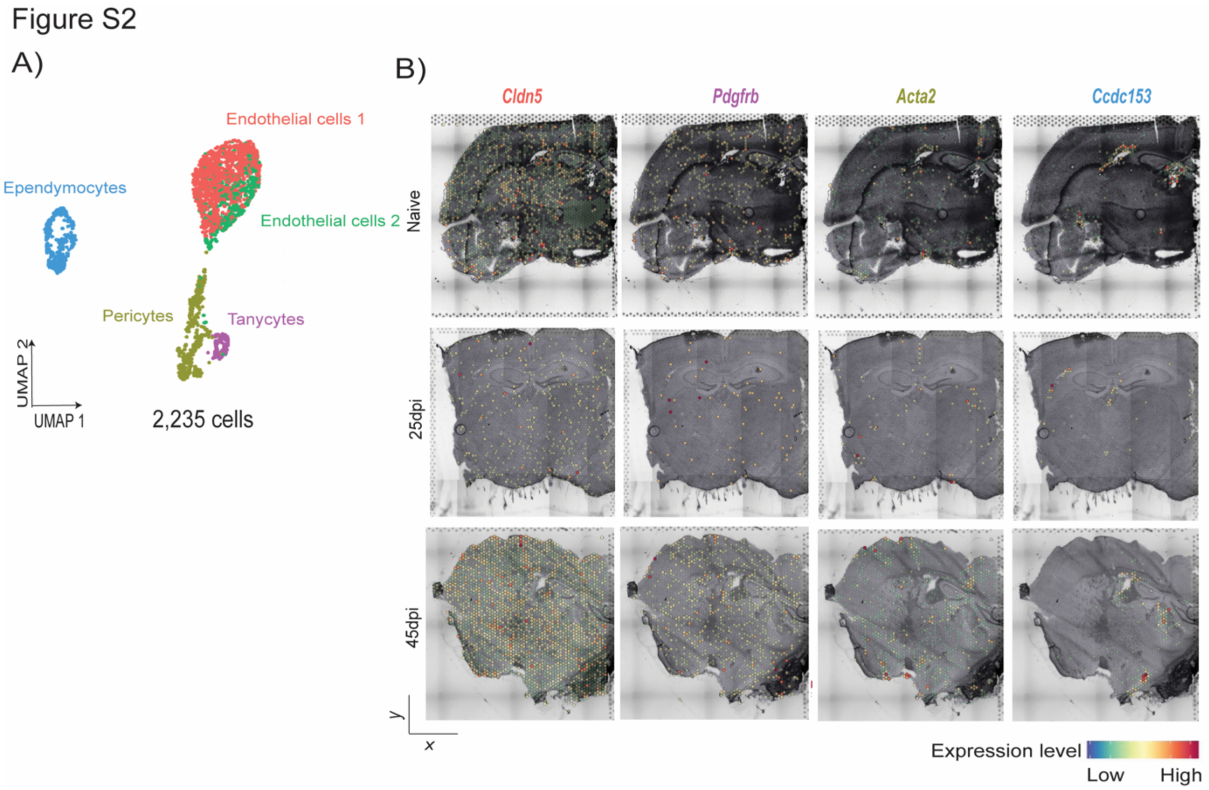

Supplementary Figure 2. Transcriptional landscape of hypothalamic astrocytes and vasculature during chronic *T. brucei* infection.

A) UMAP plot depicting the various vascular-associated cell types identified in the hypothalamic scRNAseq dataset. **B)** Spatial feature plot of putative marker genes for the various vasculature-associated cells in the 10X Visium dataset from naïve (top), 25dpi (middle), and 45dpi (bottom) coronal mouse brain sections. The relative expression level is indicated, and colour coded.

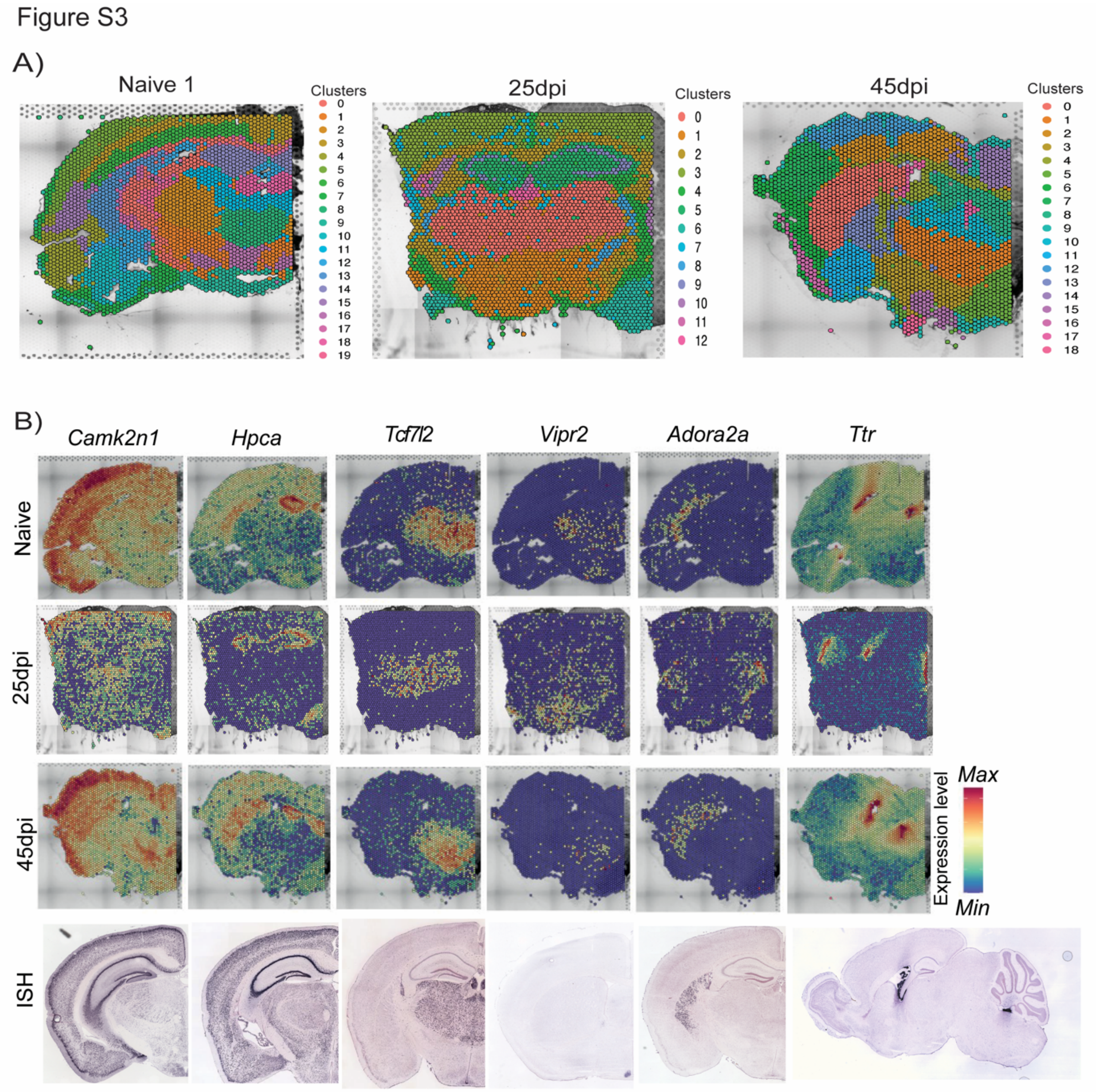

Supplementary Figure 3. Quality control of 10X Visium datasets from the mouse forebrain over the course of infection with *T. brucei*

A) Spatially resolve gene cluster analysis and dimensional reduction for naïve (left), 25dpi (middle), and 45dpi (right) 10X Visium spatial transcriptomics. **B)** Spatially resolved expression level of putative marker genes defining different anatomical brain regions in naïve (top) and 45dpi (bottom) samples. The transcriptional patters coincide with in situ hybridisation analysis of the following marker genes from the Allen Mouse brain atlas: *Camk2* (http://mouse.brain-map.org/experiment/show/79490122), *Hpca* (http://mouse.brain-map.org/experiment/show/72129291), *Tcf7l2* (http://mouse.brain-map.org/experiment/show/72339557), *Vipr2* (http://mouse.brain-

map.org/experiment/show/1104), *Adora2a* (http://mouse.brain-map.org/experiment/show/72109410), *Ttr* (http://mouse.brain-map.org/experiment/show/68632172) [107–110]. The Images were obtained from the Allen Brain Atlas (open source) and the links provided. Abbreviations: d3V, dorsal 3rd ventricle; Th, Thalamus; Ctx, Cerebral cortex; CPu, Caudoputamen; LV, Lateral ventricle; Hip, Hippocampus; Hyp, Hypothalamus.

Figure S4

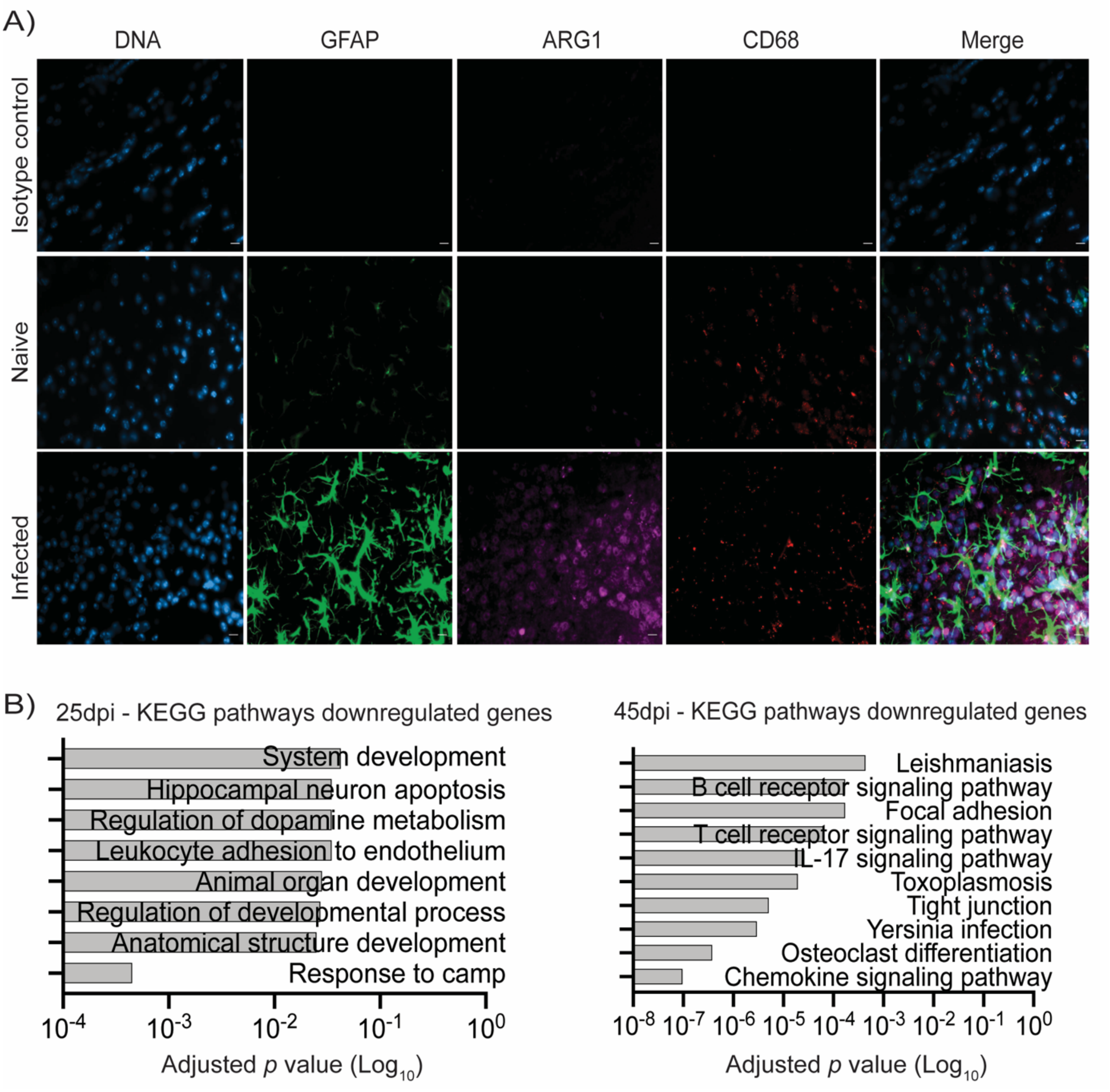

Supplementary Figure 4. Characterisation of CNS myeloid responses to T. brucei infection.

A) Imaging analysis of *Mrc1*$^+$ BAMs in coronal brain sections of naïve and 45 day-infected mice using immunofluorescence staining for the detection of CD68 (pan-microglia marker) and ARG1 (BAM specific marker). DAPI was included as nuclear staining, and GFAP as a marker for astrocyte reactivity. An IgG isotype control is also included. Scale bar = 25 µm. The results presented here are representative from two independent experiments. **B)** Examples of downregulated gene pathways overrepresented in HM 2 microglia at 25 and 45dpi and was determined using the non-parametric Wilcoxon rank sum test.

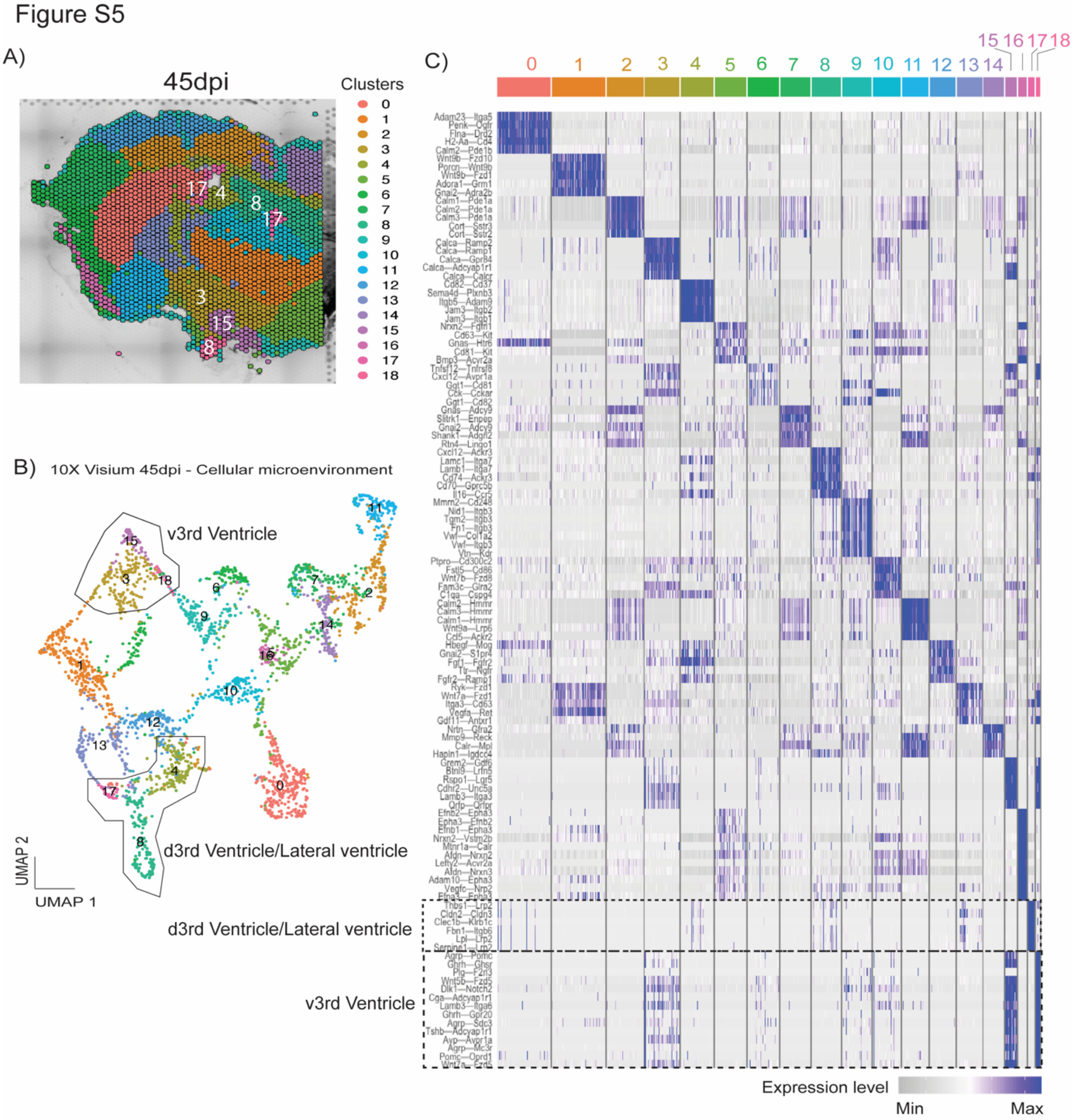

Supplementary Figure 5. Ligand-receptor interaction network in the mouse brain at 45 days post-infection.

A) Spatially resolved cluster genes for the 45dpi 10X Visium spatial transcriptomic dataset. **B)** Uniform Manifold Approximation and Projection (UMAP) plot depicting the predicted cluster-cluster interactions in the spatial context. Clusters are grouped based on their predicted ligand-receptor interaction. **C)** Heatmap representing the top 5 most significantly enriched ligand-receptor pairs in each of the spatially resolved transcriptional units in (A) and (B), clustered based on their relative expression.

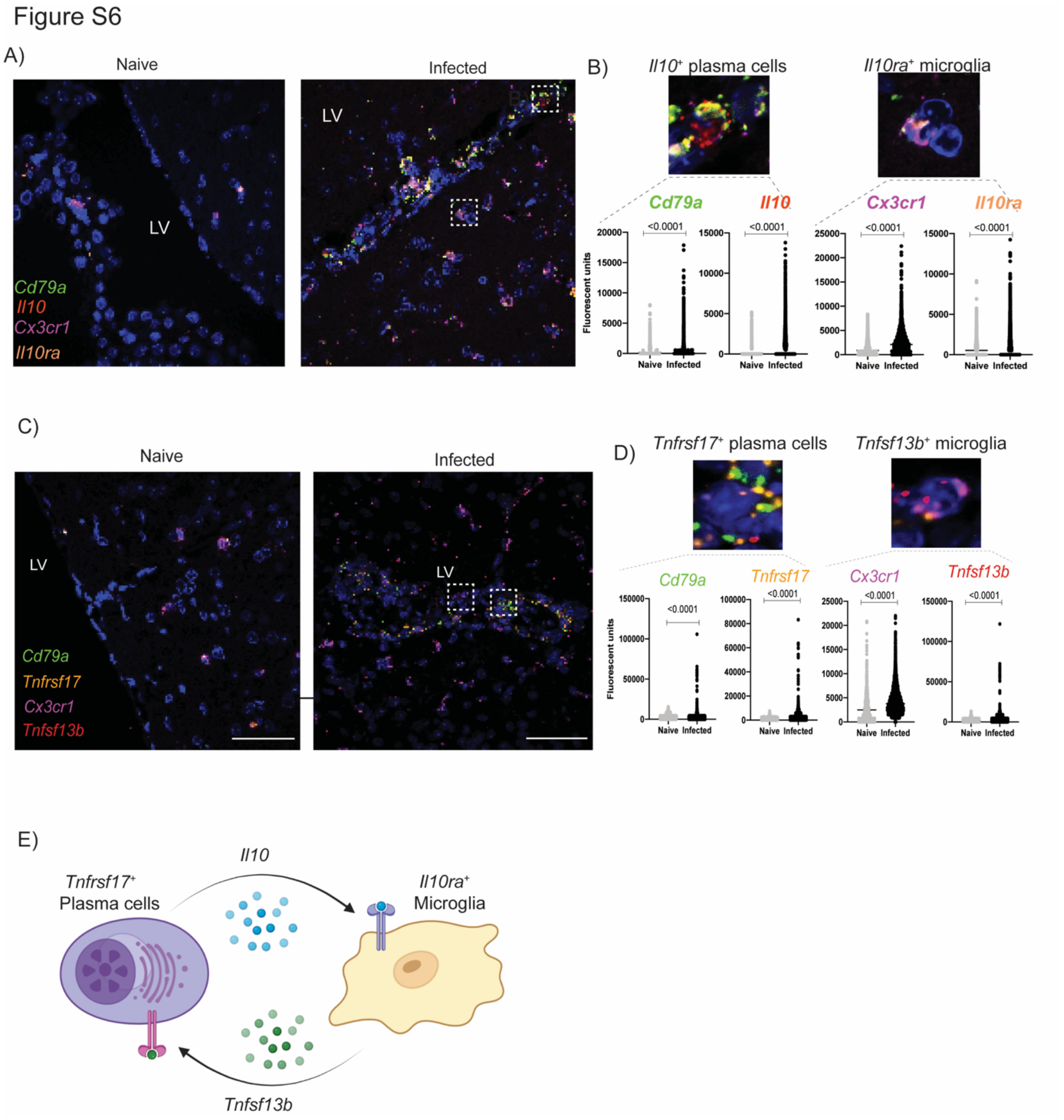

Supplementary Figure 6. Microglia – plasma cell communication mediated via *Il10* and *Tnfsf13b* signalling.

A) Chronic *T. brucei* infection induces the expression of *Il10* and *Il10ra* in B cells and Homeostatic microglia, respectively. Representative smFISH probe targeting *Cx3cr1* (purple), *Cd79a* (green), *Il10* (red), and *Il10ra* (orange) around the lateral ventricle (LV) in naïve (left) and infected (right) mouse brain coronal section. Scale bar, 25 μm. The results presented here are representative from two independent experiments. **B) Top panel**: Insets taken from the infected sample in (A), representing B cells expressing *Il10* (left) and homeostatic microglia expressing *Il10ra* (right). **Bottom panel**: Quantification of fluorescence intensity of the smFISH probes across four biological

replicates (n = 4) per experimental group (n = 1,630 nuclei from naïve samples and 3,906 nuclei from infected samples). Two-sided non-parametric Mann-Whitney test p value < 0.01 is considered significant. **C)** Chronic *T. brucei* infection induces the expression of *Tnfsf13b* and *Tnfrsf17* in homeostatic microglia and plasma cells, respectively. Representative smFISH probe targeting *Cx3cr1* (purple), *Cd79a* (green), *Tnfsf13b* (red), and *Tnfrsf17* (orange) around the lateral ventricle (LV) in naïve (left) and infected (right) mouse brain coronal section. Scale bar, 25 μm. The results presented here are representative from two independent experiments. **D) Top panel**: Insets taken from the infected sample in (A), representing plasma cells expressing *Tnfrsf17* (left) and homeostatic microglia expressing *Tnfsf13b* (right). **Bottom panel**: Quantification of fluorescence intensity of the smFISH probes across four biological replicates (n = 4) per group (n = 919 nuclei from naïve samples and 4,026 nuclei from infected samples). Two-sided non-parametric Mann-Whitney test p value < 0.01 is considered significant. **E)** Proposed microglia-plasma cell crosstalk mediated by *Il10* and *Tnfsf13b*. In this context, microglia promote plasma cell maintenance and survival via *Tnfsf13b* (BAFF) signalling, whereas plasma cells dampen pro-inflammatory responses in microglia via *Il10* signalling (created with BioRender.com).
