# Supplementary material for: Single cell and spatial transcriptomic analyses reveal microglia-plasma cell crosstalk in the brain during Trypanosoma brucei infection
Source: Nat Commun. 2022 Sep 30;13:5752. doi: 10.1038/s41467-022-33542-z (PMC9525673; doi:10.1038/s41467-022-33542-z)
Supplement: Supplementary file 3 — Description of Additional Supplementary Files [file 41467_2022_33542_MOESM3_ESM.pdf]

Description of Additional Supplementary Files

Supplementary Data 1. Characterisation of intra- and extra-ventricular trypanosomes in the mouse forebrain. Histopathology analysis from C57BL/6 female mice infected with *T. brucei brucei* Antat 1.1E were harvested at 25- and 45- days post-infection (*n* = 3-4 mice/group), fixed in 10% PFA and counterstained with the *T. brucei*-specific antibody TbHSP70. Uninfected animals (*n* = 3) were included as naïve controls. Clinical scoring on coronal sections were scored using a double-blinded approach. In addition to relative parasite localisation, H&E staining was also included to assess meningeal and parenchymal inflammation. The classification used for the scoring system is included in the table.

Supplementary Data 2. Overview of the major cell types detected in the single cell dataset at a resolution of 0.4. The total number of cells per cluster, percentages, and marker genes are also included. Marker genes were identified using the non-parametric Wilcoxon rank sum test used by the Seurat package by default.

Supplementary Data 3. Overview of the outputs generated by scCATCH and SingleR. The predicted cell identity, as well as the prediction score (where applicable), are included.

Supplementary Data 4. Overview of the vascular-associated cells detected in our hypothalamic dataset at a resolution of 0.3. The marker genes for these clusters, as well as representative UMAP plots are also included.

Supplementary Data 5. Mouse and *T. brucei* marker genes identified in the 10X Visium spatial transcriptomics datasets. Marker genes were identified using the non-parametric Wilcoxon rank sum test used by the Seurat package by default.

Supplementary Data 6. Gene Ontology analysis (Biological processes) generated using TriTrypDB and default settings. Significantly enriched pathways are defined as having an adjusted p value < 0.05 using the non-parametric Wilcoxon rank sum test.

Supplementary Data 7. Overview of the marker genes for the myeloid subclusters at a resolution of 0.3**.**

Supplementary Data 8. Marker genes detected with the integration workflow STACAS for the cells detected in the hypothalamic dataset, and the myeloid subset. Marker genes were identified using the non-parametric Wilcoxon rank sum test.

Supplementary Data 9. List of differentially expressed genes in selected myeloid subsets at 25dpi, defined having a -0.25 < Log_2Fold change >0.25 and an adjusted *p* value < 0.05 using the non-parametric Wilcoxon rank sum test used by the Seurat package by default.

Supplementary Data 10. List of differentially expressed genes in selected myeloid subsets at 45dpi, defined having a -0.25 < Log_2Fold change >0.25 and an adjusted *p* value < 0.05 using the non-parametric Wilcoxon rank sum test used by the Seurat package by default.

Supplementary Data 11. List of gene pathways identified in the IAMNP1 and IAMNP2 clusters. Significant pathways are considered those with a false discovery rate (FDR) < 0.05.

Supplementary Data 12. Marker genes detected for the T cell subsets at a resolution of 0.3.

Supplementary Data 13. Overview of the oligodendrocytes and B cells detected in our hypothalamic dataset at a resolution of 0.3. The marker genes for these clusters, cell counts, as well as representative UMAP plots are also included.

Supplementary Data 14. Quality control including mean reads per cell and median genes per cell before and after filtering out low quality cell types.

Supplementary Data 15. Overview of the spatial transcriptomics project, including total number of reads sequenced per biological replicate, the median number of genes per spot and the percentage of mappable reads to the mouse (mm10) or *T. brucei* (TREU927) genomes.
