# Supplementary material for: Single cell and spatial transcriptomic analyses reveal microglia-plasma cell crosstalk in the brain during Trypanosoma brucei infection
Source: Nat Commun. 2022 Sep 30;13:5752. doi: 10.1038/s41467-022-33542-z (PMC9525673; doi:10.1038/s41467-022-33542-z)
Supplement: Supplementary file 19 — Reporting Summary [file 41467_2022_33542_MOESM19_ESM.pdf]

nature portfolio

Corresponding author(s): Juan F. Quintana

Last updated by author(s): Aug 30, 2022

Reporting Summary

Nature Portfolio wishes to improve the reproducibility of the work that we publish. This form provides structure for consistency and transparency in reporting. For further information on Nature Portfolio policies, see our Editorial Policies and the Editorial Policy Checklist.

Statistics

For all statistical analyses, confirm that the following items are present in the figure legend, table legend, main text, or Methods section.

n/a	Confirmed	
☐	☒	The exact sample size (*n*) for each experimental group/condition, given as a discrete number and unit of measurement
☐	☒	A statement on whether measurements were taken from distinct samples or whether the same sample was measured repeatedly
☐	☒	The statistical test(s) used AND whether they are one- or two-sided<br>*Only common tests should be described solely by name; describe more complex techniques in the Methods section.*
☒	☐	A description of all covariates tested
☐	☒	A description of any assumptions or corrections, such as tests of normality and adjustment for multiple comparisons
☐	☒	A full description of the statistical parameters including central tendency (e.g. means) or other basic estimates (e.g. regression coefficient) AND variation (e.g. standard deviation) or associated estimates of uncertainty (e.g. confidence intervals)
☐	☒	For null hypothesis testing, the test statistic (e.g. *F*, *t*, *r*) with confidence intervals, effect sizes, degrees of freedom and *P* value noted<br>*Give P values as exact values whenever suitable.*
☒	☐	For Bayesian analysis, information on the choice of priors and Markov chain Monte Carlo settings
☒	☐	For hierarchical and complex designs, identification of the appropriate level for tests and full reporting of outcomes
☒	☐	Estimates of effect sizes (e.g. Cohen's *d*, Pearson's *r*), indicating how they were calculated

Our web collection on statistics for biologists contains articles on many of the points above.

Software and code

Policy information about availability of computer code

Data collection	Single cell RNA sequencing was conducted on an Illumina Novaseq 6000 sequencers by Glasgow polyomics. Spatial RNA sequencing was conducted on a NextSeq 550 Illumina instrument at Glasgow Polyomics. Fastq sequence files were de-multiplexed, aligned, and annotated using a reference combined mouse (mmu10; https://www.ncbi.nlm.nih.gov/assembly/GCF_000001635.20/) and T. brucei 927 (TREU927; https://www.ebi.ac.uk/genomes/CH464491.html) reference transcriptome built by Glasgow polyomics and and Cell Ranger software (v6.0.0; single cell RNA sequencing) or Space Ranger (v1.2.2; spatial RNA sequencing) softwares. Gene expression was counted using unique molecular identifier barcodes, and gene-cell matrices were constructed. FACS DIVA software (v9.0) was used for acquisition of flow cytometry data.
Data analysis	The following R packages were used to analyse the single cell and spatial sequencing dataset: R (v4.2.1), Seurat (v4.1.0), sctransform (v0.3.3), seater (v1.22.0), STACAS (vl.1.0), scCATCH (v2.1), SingleR (v1.8.1), nichenetr (v1.1.0), dplyr (vl.0.7), RColorBrewer (vl.1.2), ggplot (v3.3.5). Fiji v2 was used for image analysis. FlowJo (v10.8.2) (BD) were used for analysis of flow cytometry data. Code used to perform analysis described can be accessed at Zenodo (https:// zenodo.org/record/6387555#.YkW3tC8wlnk).

For manuscripts utilizing custom algorithms or software that are central to the research but not yet described in published literature, software must be made available to editors and reviewers. We strongly encourage code deposition in a community repository (e.g. GitHub). See the Nature Portfolio guidelines for submitting code & software for further information.

Data

Policy information about availability of data

All manuscripts must include a data availability statement. This statement should provide the following information, where applicable:

- Accession codes, unique identifiers, or web links for publicly available datasets
- A description of any restrictions on data availability
- For clinical datasets or third party data, please ensure that the statement adheres to our policy

The data generated in this study have been deposited in the Gene Expression Omnibus database under accession code GSE200642 (https://www.ncbi.nlm.nih.gov/geo/query/acc.cgi?acc=GSE200642). The processed transcript count data and cell metadata generated in this study are available at Zenodo (https://zenodo.org/record/6387555#.YwkaFi8w1nk)34. The flow cytometry data generated in this study are provided in the Supplementary Information/Source Data file.
Additional data and files can also be sourced via Supplementary Tables. Source data are provided with this paper and the single cell dataset can be explored in this link: https://cellatlas-cxg.mvls.gla.ac.uk/tbrucei_brain/

Human research participants

Policy information about studies involving human research participants and Sex and Gender in Research.

Reporting on sex and gender	No human samples or subjects were used in this study
Population characteristics	No human samples or subjects were used in this study
Recruitment	No human samples or subjects were used in this study
Ethics oversight	No human samples or subjects were used in this study

Note that full information on the approval of the study protocol must also be provided in the manuscript.

Field-specific reporting

Please select the one below that is the best fit for your research. If you are not sure, read the appropriate sections before making your selection.

☒ Life sciences ☐ Behavioural & social sciences ☐ Ecological, evolutionary & environmental sciences

For a reference copy of the document with all sections, see nature.com/documents/nr-reporting-summary-flat.pdf

Life sciences study design

All studies must disclose on these points even when the disclosure is negative.

Sample size	The maximum sample size was used for all the transcriptomics experiments presented in this study, within the limitations of the technology implemented (~1s,ooo cells per experiment, and 4 tissue sections per spatial transcriptomic slide). For in vivo experiments, we used a sample size of 5 animals per experimental condition. This sample size is based on measures of variance from historical data in the laboratory, and is enough to detect differences between experimental and control groups at a 5% significance level and 90% power.
Data exclusions	No data were excluded from the analysis
Replication	All experiments were conducted using independent biological replicates (at least n = 2 for single cell transcriptomics). The data obtained from the single cell experiments were succesfully replicated in independent experiments (e.g., regulatory B cells during chronic infection). Spatial transcriptomics datasets were not replicated due to costs and logistics, but were independently validated using in situ hybridisation approaches. All flow cytometry measurements were conducted in at least two independent experiments with similar results. The analysis of supernatant obtained from brain-dwelling B cells was conducted from several mice and repeated twice.
Randomization	Randomisation of scRNAseq or spatial transcriptomics was not appropriate as both naive and infected animals were processed in parallel and required additional labelling for downstream analysis (e.g., flow cytometry analysis, H&E analysis). Randomisation of in vivo experiments was not appropriate as the infected animals required to be closely monitored for the development of clinical adverse effects. Animals used for flow cytometry and imaging analysis described here as validation for single cell and spatial transcriptomics were randomly allocated into cages.
Blinding	Blinding of scRNA was not appropriate as data processing and analysis need to be carried out appropriately and differentially for biological between experimental conditions. Measurements associated with parasitaemia and clinical scoring were conducted by a trained scientist blinded to the study. For flow cytometry analysis and immunohistochemistry, blinding was not carried out in order to allow inclusion of appropriate measurements of background intensity and signal adjustments during acquisition.

Reporting for specific materials, systems and methods

We require information from authors about some types of materials, experimental systems and methods used in many studies. Here, indicate whether each material, system or method listed is relevant to your study. If you are not sure if a list item applies to your research, read the appropriate section before selecting a response.

Materials & experimental systems

n/a	Involved in the study
☐	☒ Antibodies
☐	☒ Eukaryotic cell lines
☒	☐ Palaeontology and archaeology
☐	☒ Animals and other organisms
☒	☐ Clinical data
☒	☐ Dual use research of concern

Methods

n/a	Involved in the study
☒	☐ ChIP-seq
☐	☒ Flow cytometry
☒	☐ MRI-based neuroimaging

Antibodies

Antibodies used

The following anti-mouse antibodies were used for flow cytometry experiments: Fixable viability dye (Thermo, 65-0865-14), CD45-PE (Biolegend, 147712, clone I3/2.3, 2 micrograms/mouse), CD45-Brilliant Violet 421 (Biolegend, 103133, clone 30-F11, 1/400), CD19-Brilliant Violet 711, 115555, clone 6D5, 1/400), B220-Alexa Fluor 488 (Biolegend, 103225, clone RA3-6B2, 1/400), CD138-PE Dazzle 594 (Biolegend, 142527, clone 281-2, 1/400), CD11b-Alexa Fluor 488 (Biolegend, 101219, clone M1/70, 1/400), BAFF-APC (Biolegend, 366507, clone 1D6, 1/400), CD206-Brilliant Violet 711 (Biolegend, 141727, clone C068C2, 1/400), O4-APC (Miltenyi, 130-119-982, Clone REA576, 1/800), ACSA-PE (Miltenyi, 130-123-284, clone IH3-18A3, 1/800). The following antibodies were used for imaging: Arginase 1-Alexa Fluor 647 (Cell Signalling, 43279S, clone D4E3M, 1/100), CD68-PE (Miltenyi, 130-114-651, clone REA886, 1/50), GFAP-Alexa Fluor 488 (Santa Cruz, sc-33673, clone 2E1, 1/100).

Validation

All antibodies for flow cytometry were used at the recommended concentration by the manufacturer and tested in pilot titration experiments. The antibodies used for imaging were used exactly as recommended by the manufacturer, including the concentration.

Eukaryotic cell lines

Policy information about cell lines and Sex and Gender in Research

Cell line source(s)

Murine microglia cell line BV2 were kindly gifted by Dr. Marieke Pingen, University of Glasgow. This cell line is commercially available on the can be purchased from the American Type Culture Collection (Cat. Number CRL-2467TM). Cells were cultured in DMEM medium (Sigma) supplemented with 10% foetal bovine serum (FBS) (Sigma) and 1000 IU/ml penicillin, and 100 mg/ml streptomycin, and maintained at 37oC and 5% CO2.

Authentication

These cells were not authenticated, but they display a robust pro-inflammatory state when exposed to bacterial LPS, as expected for this microglia cell line.

Mycoplasma contamination

No mycoplasma testing was performed.

Commonly misidentified lines
(See ICLAC register)

No commonly misidentified cell lines were used in this study

Animals and other research organisms

Policy information about studies involving animals; ARRIVE guidelines recommended for reporting animal research, and Sex and Gender in Research

Laboratory animals

Adult female (6-8 week old) female C57BL/6J (WT) mice were purchased from Jackson Laboratories (JAX stock 000664). Mice were age and weight-matched within experiments. Animals were housed on a 12 h light-dark cycle and fed ad libitum. Room temperature was between 20-24C and humidity was between 50-70%.

Wild animals

No wild animals were used in this study

Reporting on sex

Only female mice were used in this study. Historical data in the laboratory suggests that both sexes respond similarly to infection by Trypanosoma brucei.

Field-collected samples

No field samples were used in this study

Ethics oversight

All animal experiments were approved by the University of Glasgow Ethical Review Committee and performed in accordance with the home office guidelines, UK Animals (Scientific Procedures) Act, 1986 and EU directive 2010/63/EU. All experiments were conducted under SAPO regulations and UK Home Office project licence number PC8C3B25C to Dr. Jean Rodger. The in vivo work related to the single cell and spatial transcriptomic experiments were conducted at 25- and 45-days post-infection (dpi) and correlated with

increased clinical scores and procedural severity. Subsequent in vivo experiments for experimental validation (flow cytometry and imaging) were terminated earlier in line with ethical recommendations from the veterinary team at the University of Glasgow.

Note that full information on the approval of the study protocol must also be provided in the manuscript.

Flow Cytometry

Plots

Confirm that:

☒ The axis labels state the marker and fluorochrome used (e.g. CD4-FITC).

☒ The axis scales are clearly visible. Include numbers along axes only for bottom left plot of group (a 'group' is an analysis of identical markers).

☒ All plots are contour plots with outliers or pseudocolor plots.

☒ A numerical value for number of cells or percentage (with statistics) is provided.

Methodology

Sample preparation	To discriminate circulating versus brain-resident immune cells, we performed intravascular staining of peripheral CD45+ immune cells as previously reported 31. Briefly, a total of 2 ug of anti-CD45-PE antibody (in 100 ul of lX PBS) was injected intravenously 3 minutes prior culling. Mice were euthanised as described above and transcardially perfused with ice-cold 0.025% (wt/vol) EDTA in lX PBS. Whole brain samples were collected and placed on ice-cold lX HBSS (lnvitrogen) and processed as recently described 32. Whole brain specimens were minced and digested using the Adult Brain dissociation kit (Miltenyi) for 30min at 37°C, following manufacturer's recommendations. The digested tissue was gently pressed through 70 µm nylon mesh cell strainers to obtain a single cell suspension. The cell suspension was cleaned up and separated from myelin debris using a Percoll gradient, as previously reported. For flow cytometry analysis, single cell suspensions were resuspended in ice-cold FACS buffer (2mM EDTA, SU/ml DNAse I, 25mM HEPES and 2.5% Foetal calf serum (FCS) in lX PBS) and stained for extracellular markers. The list of flow cytometry antibodies used in this study were obtained from Biolegend and are presented in the table below. Samples were run on a flow cytometer LSRFortessa (BD Biosciences) and analysed using FlowJo software version 10 (Treestar). For intracellular staining, single-cell isolates from brain or draining lymph nodes were stimulated as above in lscove's modified Dulbecco's media (supplemented with lx non-essential amino acids, SOU/ml penicillin, SOug/ml streptomycin, 50µM β-mercaptoethanol, lmM sodium pyruvate and 10%FBS. Gibco). Cells were then permeabilized with a Foxp3/Transcription Factor Staining Buffer Set (eBioscience) and stained for 30min at 4°C.
Instrument	BD Fortessa Flow Cytometre (BD Biosciences)
Software	FACS Diva software (v9.0) and FlowJo (vl0.8.2) (BD).
Cell population abundance	25 µ of liquid compensation beads (BD Biosciences, 335925) were added to allow for quantification of absolute cell numbers.
Gating strategy	Unstained controls and isotype controls were used to identify background staining levels and determine gate placement. Doublets were excluded based on linearity of FSC-A and FSC-H. From singlets, live cells were identified as the Zombie NIR- population. Resident CD45+ lymphocytes were discriminated from circulating CD45+ lymphocytes on the basis of CD45-PE staining (PE+= circulating; PE- resident). For the gating of downstream cell types, we focused only on resident CD45+ lym hocytes. Plasma cells were designated CD19-CD138+ cells, and microglia were designated as CD45LowCDllb+ cells.

☒ Tick this box to confirm that a figure exemplifying the gating strategy is provided in the Supplementary Information.
